# Deep Learning Classification of Tuberculosis Chest X-rays

**DOI:** 10.7759/cureus.41583

**Published:** 2023-07-08

**Authors:** Kartik K Goswami, Rakesh Kumar, Rajesh Kumar, Akshay J Reddy, Sanjeev K Goswami

**Affiliations:** 1 College of Medicine, California Northstate University College of Medicine, Elk Grove, USA; 2 Pulmonary and Critical Care Medicine, Stockton Pulmonary Doctors, Stockton, USA; 3 Nursing, St. Joseph Medical Center, Stockton, USA; 4 Medicine, California University of Science and Medicine, Colton, USA; 5 Pulmonary and Critical Care Medicine, St. Joseph Medical Center, Stockton, USA

**Keywords:** disease, internal medicine, medicine, chest x-ray (cx-ray), x-ray, imaging, infectious disease, tuberculosis, tb, pulmonology

## Abstract

Background

Tuberculosis (TB) is an infectious disease caused by the bacterium *Mycobacterium tuberculosis*. It primarily affects the lungs but can also affect other organs, such as the kidneys, bones, and brain. TB is transmitted through the air when an infected individual coughs, sneezes, or speaks, releasing tiny droplets containing the bacteria. Despite significant efforts to combat TB, challenges such as drug resistance, co-infection with human immunodeficiency virus (HIV), and limited resources in high-burden settings continue to pose obstacles to its eradication. TB remains a significant global health challenge, necessitating accurate and timely detection for effective management.

Methods

This study aimed to develop a TB detection model using chest X-ray images obtained from Kaggle.com, utilizing Google’s Collaboration Platform. Over 1196 chest X-ray images, comprising both TB-positive and normal cases, were employed for model development. The model was trained to recognize patterns within the TB chest X-rays to efficiently recognize TB within patients in order to be treated in time.

Results

The model achieved an average precision of 0.934, with precision and recall values of 94.1% each, indicating its high accuracy in classifying TB-positive and normal cases. Sensitivity and specificity values were calculated as 96.85% and 91.49%, respectively. The F1 score was also calculated to be 0.941. The overall accuracy of the model was found to be 94%.

Conclusion

These results highlight the potential of machine learning algorithms for TB detection using chest X-ray images. Further validation studies and research efforts are needed to assess the model's generalizability and integration into clinical practice, ultimately facilitating early detection and improved management of TB.

## Introduction

Tuberculosis (TB) remains one of the most prevalent infectious diseases globally, posing a significant public health challenge, currently treated with an array of drugs and vaccines [1]. Caused by the bacterium *Mycobacterium tuberculosis*, TB primarily affects the lungs but can also affect other organs, leading to severe morbidity and mortality if left untreated [2,3]. Before the COVID-19 pandemic, TB was the leading cause of death from a single infectious agent, ranking above both human immunodeficiency virus (HIV) and acquired immune deficiency syndrome (AIDS) in terms of mortality [4]. According to the World Health Organization (WHO), TB was responsible for approximately 1.4 million deaths and 10 million new cases worldwide in 2019 [5]. TB deaths have increased because of reduced access to care. TB deaths have been increasing recently, with 2020 having 1.5 million TB deaths worldwide which was the first year-over-year increase since 2005 [6]. 

The complex nature of TB, coupled with its ability to spread through airborne droplets, makes early detection crucial for effective disease management and prevention of transmission [7]. Traditional diagnostic methods, such as sputum smear microscopy and culture, have limitations in terms of sensitivity, speed, and accessibility, particularly in resource-limited settings [8].

In recent years, medical imaging techniques, such as chest X-ray imaging, have emerged as valuable tools for TB diagnosis [8]. Chest X-rays provide detailed anatomical information and can reveal characteristic radiological patterns associated with TB, such as infiltrates, cavitations, and nodules [7,8]. However, the interpretation of chest X-ray images by human experts is subjective and can be influenced by variations in expertise and experience.

Advances in machine learning and deep learning algorithms offer a promising avenue for improving TB diagnosis by leveraging the power of artificial intelligence (AI) to analyze chest X-ray images. These algorithms can learn intricate patterns and features associated with TB, leading to more accurate and consistent detection.

In this investigation, we sought to develop a TB detection model using chest X-ray images. The successful development of a reliable and accurate TB detection model could revolutionize TB diagnosis by providing a standardized and objective tool for healthcare professionals. Such models could support early detection, prompt initiation of treatment, and implementation of effective TB control measures. Moreover, the integration of AI-based TB detection models into clinical workflows could particularly benefit regions with limited access to expert radiologists and healthcare resources.

This study investigates the potential of machine learning algorithms in improving TB diagnosis using chest X-ray images. By leveraging AI technology, we aim to develop a robust TB detection model that can enhance the accuracy and efficiency of TB diagnosis, ultimately contributing to the global efforts to combat this persistent infectious disease.

## Materials and methods

This study aimed to develop a TB detection model using chest X-ray images obtained from datasets on Kaggle.com [9]. A dataset of over 1196 chest X-ray images, including 633 TB-positive chest X-rays and 563 normal cases, was utilized for model development. The images were in digital format (e.g., JPEG, PNG) and were labeled accordingly.

To preprocess the data, the TB and normal images were organized into separate folders. Each image was visually inspected to ensure proper labeling and quality. No additional preprocessing, such as image resizing or augmentation, was performed.

**Figure 1:**
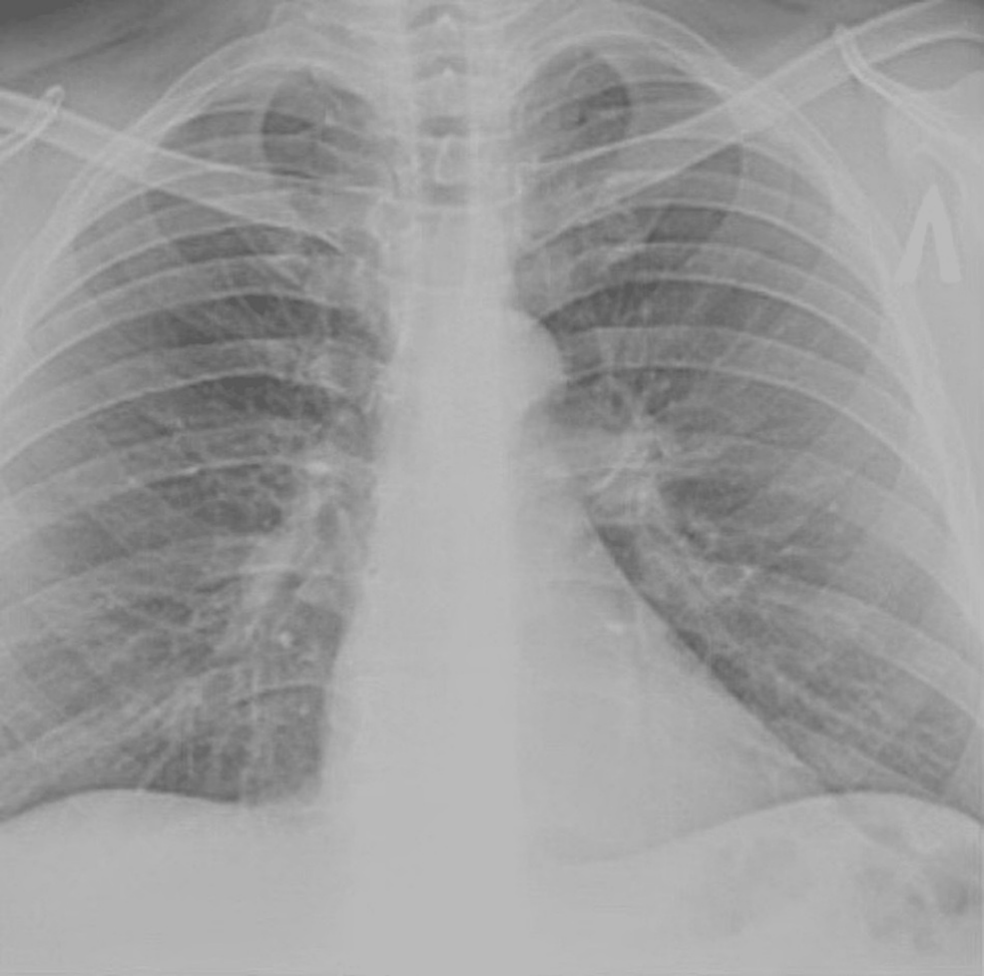
TB chest X-ray dataset example TB, tuberculosis.

For model development, Google’s Collaboration Platform was employed. The deep learning algorithm, using CNN methods, was trained to learn the patterns and features associated with TB-positive X-rays using the labeled chest X-ray dataset, as seen in Figure 1. 

This was then trained by using 80% of the images to train the model, 10% of the images to validate the model, and another 10% of the images to test the model. The assignments in this study were all randomized. No external datasets were used in this study.

**Figure 2:**
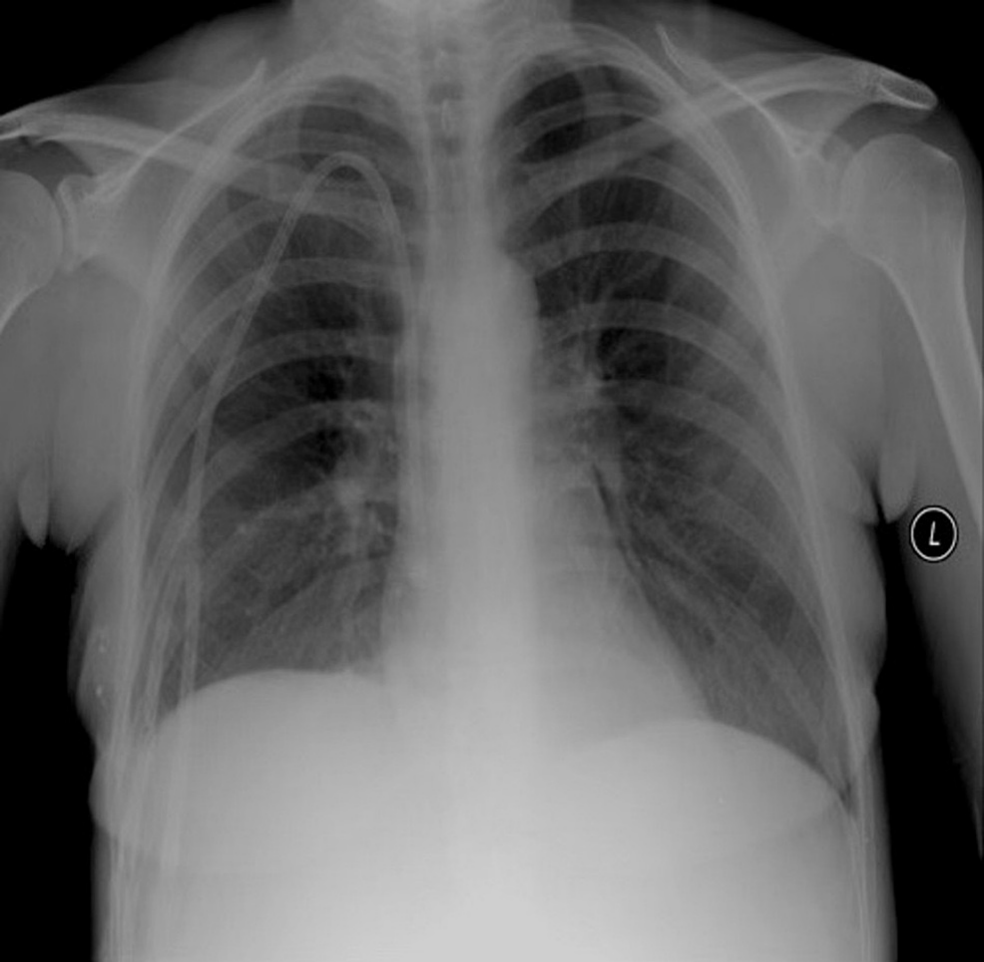
Normal chest X-ray dataset example

The trained model was evaluated using a separate test set of chest X-ray images. Its performance was assessed based on its ability to accurately identify TB-positive X-rays and distinguish them from normal X-rays, as seen in Figure 2.

Evaluation metrics, including accuracy, precision, recall, and confusion matrix, were calculated to quantify the model's performance.

The model was constructed using a software tool supported by Google. The software tools used included Google’s Collaboration Platform, Python, and deep learning frameworks such as TensorFlow and PyTorch.

## Results

**Figure 3:**
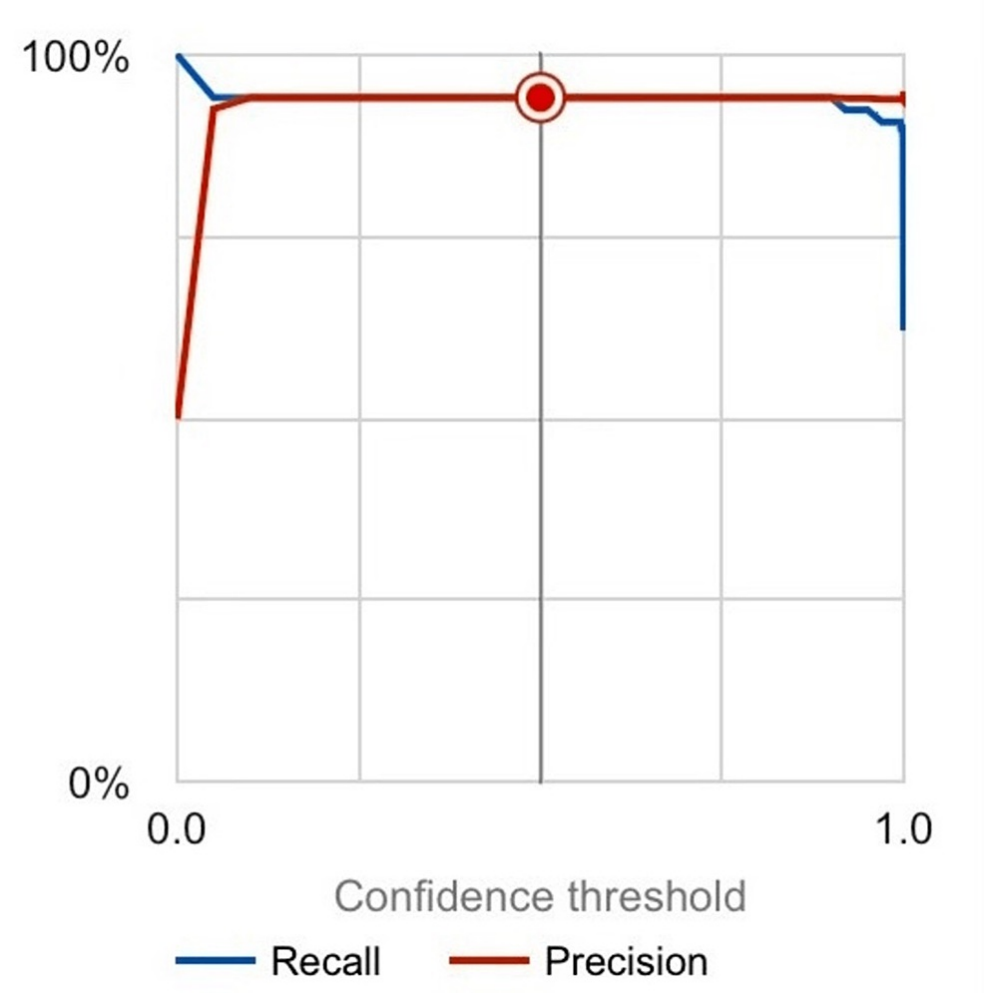
AUC graph showing the recall and precision of the TB model AUC, area under the curve; TB, tuberculosis.

The TB detection model developed using Google’s Collaboration Platform exhibited promising performance. The model achieved an area under the curve (AUC) of 0.934, indicating its ability to accurately classify TB-positive and normal chest X-ray images. Both precision and recall values were found to be 94.1%, highlighting the model's high accuracy in correctly identifying TB cases as seen in Figure 3.

A possible limitation from a high recall means that the model could be overfitting, where the machine learning algorithm memorizes the data rather than learning from patterns. This could lead to recall rates near 100, which would mean the model is just memorizing the pictures and is not able to reliably predict future occurrences since it did not learn.

**Figure 4:**
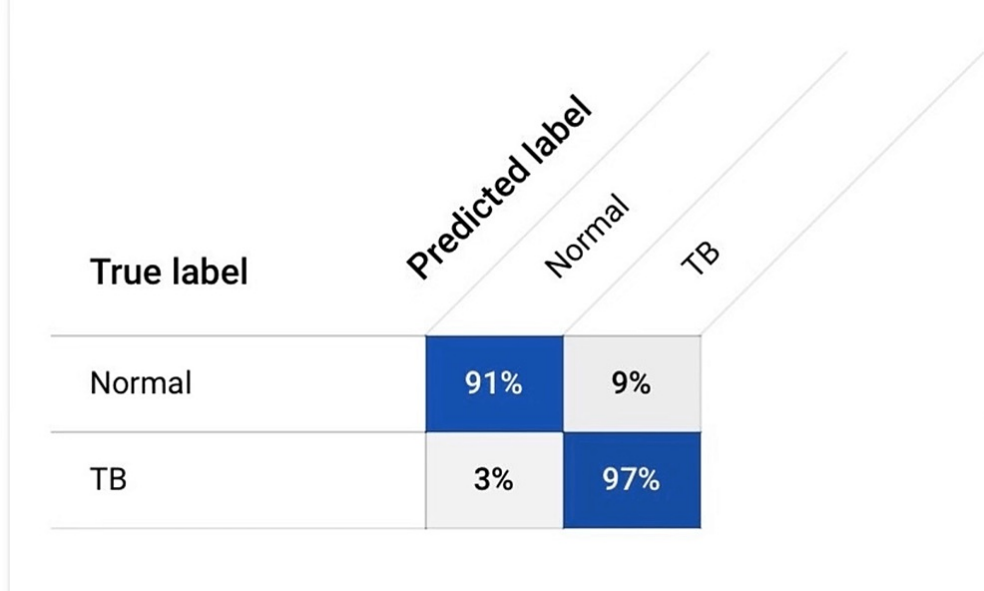
Confusion matrix TB, tuberculosis.

To further evaluate the model's performance, statistics of sensitivity, specificity, and overall accuracy were calculated using the provided values. The values were calculated by finding the true positive, true negative, false positive, and false negative from the Confusion matrix in Figure 4.

The sensitivity, also known as the true positive rate, was determined to be 96.85%. This indicates that the model correctly identified 96.85% of actual TB-positive cases. The specificity, or true negative rate, was calculated to be 91.49%, reflecting the model's proficiency in correctly classifying normal cases.

Furthermore, the overall accuracy of the model was determined to be 94%. This value represents the proportion of all cases, both TB-positive and normal, that were correctly classified by the model. These results highlight the model's effectiveness in distinguishing between TB and normal chest X-ray images.

It is important to note that the experiments were conducted using Google’s Collaboration Platform on May 22. These results demonstrate the model's high sensitivity in identifying TB-positive cases and its specificity in recognizing normal cases. The achieved accuracy underscores the model's overall performance in accurately classifying TB cases.

## Discussion

TB remains a significant global health concern, and early and accurate detection is crucial for effective management and control [10]. In this study, we aimed to develop a TB detection model using chest X-ray images obtained from Kaggle.com’s TB chest X-ray dataset, leveraging Google’s Collaboration Platform. The model achieved an average precision of 0.934, with precision and recall values of 94.1% each, indicating its high accuracy in classifying TB-positive and normal cases.

The results obtained from this study highlight the potential of machine learning and deep learning algorithms in the field of TB detection. Chest X-ray imaging is a widely accessible and cost-effective diagnostic tool for TB, making it an ideal modality for training and evaluating detection models. By leveraging a large dataset of over 1196 chest X-ray images, including both TB-positive and normal cases, we were able to develop a model that demonstrated remarkable performance.

The sensitivity, or true positive rate, of the developed model was calculated to be 96.85%. This indicates that the model successfully identified a significant proportion of actual TB-positive cases, minimizing false negatives. The specificity, or true negative rate, was determined to be 91.49%, reflecting the model's ability to accurately classify normal cases and avoid false positives. These results indicate the model's efficacy in correctly identifying both TB-positive and normal chest X-ray images.

The high accuracy of the model, as reflected in the overall accuracy of 94%, suggests its potential for aiding in the early detection of TB. Early diagnosis is crucial for initiating timely treatment and curbing the transmission of the disease [10]. The development of accurate TB detection models can assist healthcare professionals in making informed decisions and improving patient outcomes.

It is important to acknowledge the limitations of this study. First, the study relied on the use of publicly available chest X-ray images from Kaggle.com, which may not represent the full spectrum of TB cases or account for the variations in imaging quality encountered in real-world clinical settings. Additionally, the performance of the model may be influenced by factors such as the size and diversity of the dataset, as well as the choice of deep learning algorithms and hyperparameters. This model is also limited in its scope of diagnosing TB as it can only process data from chest X-ray scans, and it has not been built to take into account data from TB blood or skin tests. Perhaps future investigations could work to build such a model. 

Furthermore, while the model achieved high accuracy, it is essential to consider its performance in real-world clinical settings and evaluate its generalizability across different populations and imaging systems. Another limitation of a high-accuracy model is that it may have been overfitting and not learning from the patterns within the given datasets. Further validation studies using diverse and larger datasets, including external validation with independent cohorts, are needed to assess the robustness and reliability of the model.

This study demonstrated the potential of machine learning and deep learning algorithms in developing an accurate TB detection model using chest X-ray images. The model exhibited high precision, recall, sensitivity, specificity, and overall accuracy, suggesting its utility in distinguishing between TB-positive and normal cases. Further research and validation efforts are necessary to enhance the model's generalizability and facilitate its integration into clinical practice, ultimately aiding in the early detection and improved management of TB which would lead to faster treatment and better long-term outcomes.

TB is a complex infectious disease caused by *Mycobacterium tuberculosis*, primarily affecting the lungs but capable of spreading to other organs. It remains a major global health burden, causing significant morbidity and mortality. Chest X-ray imaging plays a vital role in the diagnosis of pulmonary TB, assisting clinicians in identifying characteristic radiological features such as infiltrates, cavitations, and nodules [8,10]. Early diagnosis of TB is crucial for initiating appropriate treatment and preventing the transmission of the disease [11].

By harnessing the power of machine learning and deep learning algorithms, our study contributes to the ongoing efforts to improve TB diagnosis. It should be noted that this model alone can not be used for an accurate diagnosis of TB, but other findings such as blood and skin tests are also needed to fully confirm the diagnosis; however, this model is still useful in that it can expedite the portion of the diagnostic process that involved the utilization of chest X-ray scans. 

The application of machine learning algorithms to chest X-ray images shows promise for advancing TB diagnosis. The model developed in this study demonstrated high accuracy and performance metrics, suggesting its potential as a valuable tool in TB detection. However, further research, validation, and clinical implementation are warranted to ensure the model's effectiveness in diverse populations and real-world clinical settings. Collaborative efforts between researchers, clinicians, and policymakers are necessary to realize the full potential of these models in combating TB and reducing its global impact.

## Conclusions

In conclusion, this study highlights the potential of machine learning and deep learning algorithms in improving TB diagnosis using chest X-ray images. The developed TB detection model demonstrated high accuracy and performance metrics, showcasing its ability to effectively classify TB-positive and normal cases. The integration of such models into clinical practice has the potential to enhance early detection, facilitate prompt treatment initiation, and improve TB control measures. However, further research, validation, and implementation efforts are necessary to ensure the model's generalizability and efficacy in diverse populations and real-world clinical settings. Collaborative efforts between researchers, clinicians, and policymakers are crucial to harnessing the full potential of these technological advancements in combating TB and reducing its global impact.
